# Rhizosphere triangular warfare: mechanistic insights for plant- beneficial microbe- pathogen interactions

**DOI:** 10.3389/fpls.2026.1942866

**Published:** 2026-09-30

**Authors:** Pratiksha Singh, Rajesh Kumar Singh, Dao-Jun Guo, Mohini Prabha Singh, Bao-Qing Zhang, Xiu-Peng Song, Sudhir Kumar Upadhyay, Yang-Rui Li

**Affiliations:** 1 School of Agriculture Science, LNCT University, Bhopal, Madhya Pradesh, India; 2 Key Laboratory of Sugarcane Biotechnology and Genetic Improvement (Guangxi), Ministry of Agriculture and Rural Affairs/Guangxi Key Laboratory of Sugarcane Genetic Improvement/Sugarcane Research Institute, Guangxi Academy of Agricultural Sciences/Sugarcane Research Centre, Chinese Academy of Agricultural Sciences, Nanning, China; 3 Department of Research and Development, Agromaxx Industry, Bhopal, Madhya Pradesh, India; 4 Key Laboratory of Hexi Corridor Resources Utilisation of Gansu, Hexi University, Zhangye, China; 5 Department of Plant Breeding, Punjab Agricultural University, Ludhiana, India; 6 Research and Development Cell, Lovely Professional University, Punjab, India

**Keywords:** effector-triggered immunity, induced systemic resistance (ISR), pattern-triggered immunity (PTI), plant-microbe-pathogen interactions, rhizosphere triangular warfare

## Abstract

The rhizosphere is a living physical space that is constantly evolving and is characterized by a complex interplay between beneficial microbes, pathogens, and plants, mediated by molecular and ecological interactions. These three-way relationships constitute a rhizosphere “triangular warfare system,” which controls plant health, nutrient uptake, disease resistance, and plant resilience. This review provides a mechanistic overview of the molecular, metabolic and ecological processes underlying these tripartite interactions. We discuss how plants integrate pattern-triggered immunity (PTI), effector-triggered immunity (ETI), microbiome exudation, and hormone signaling to select beneficial microbes to exclude pathogen entry. Beneficial microbes deploy volatile organic compounds, induction of systemic resistance (ISR), secretion systems, phytotoxins, siderophores, lytic enzymes, and microbiome-disrupting strategies to enhance plant fitness, whereas pathogens use virulence effectors, lytic enzymes, secretion systems, phytotoxins, siderophores, and microbiome-disrupting strategies to overcome host defenses and restructure rhizosphere communities. Recent advances in multi-omics have revealed the intricate signaling networks, metabolic interactions, and microbial competition shaping these relationships under various environmental conditions. We further explore emerging opportunities in microbiome engineering, synthetic microbial consortia and disease-suppressive soil management for sustainable crop protection. This review synthesizes existing mechanistic knowledge and introduces the concept of ‘rhizosphere triangular warfare’ as a stepping stone toward the next generation of microbiome-based agriculture and precision rhizosphere engineering under environmental changes.

## Graphical Abstract

**Figure:**
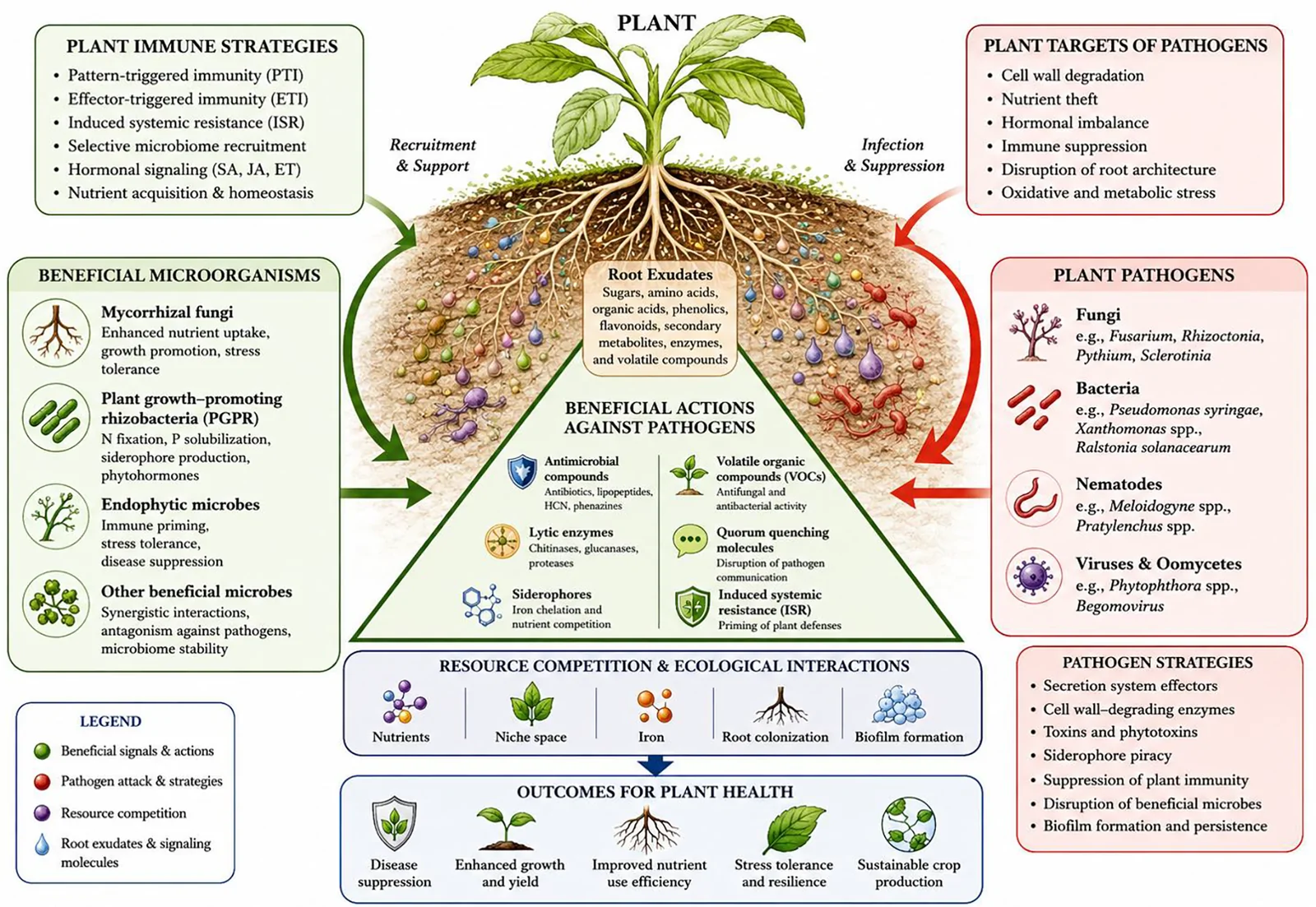

## Highlights

- Introduces a mechanistic framework for rhizosphere triangular warfare.
- Integrates plant immunity, beneficial microbes, and pathogen strategies.
- Discusses root exudate-mediated microbiome assembly and disease suppression.
- Develops multi-omics for decoding rhizosphere signaling networks.
- Explores precision microbiome engineering for sustainable crop protection.

## 1 Introduction

One of the most fertile microhabitats on earth is the rhizosphere, a zone surrounding plant roots where chemical signaling, metabolic activity and microbial diversity are at a peak (Jamil et al., 2022; Philippot et al., 2013). In this dynamic setting, plants, beneficial microbes, and pathogens continuously interact at the molecular and ecological levels, which help to control plant growth, nutrient uptake, immunity, and disease development (Adedayo and Olorunkosebi, 2025; Upadhyay, 2026a). These dynamic three-way interaction between plants, beneficial microbes, and pathogenic microorganisms inside the rhizosphere is intertwined, forming a “rhizosphere triangular warfare”; cooperation and competition determine plant health, soil fertility, and ecosystem sustainability (Li et al., 2021).

Plants actively attract beneficial microbes by releasing exudates containing root metabolites such as sugars, amino acids, organic acids, phenolics, flavonoids and secondary metabolites that act as nutrient sources and signaling molecules influencing the microbial community of the rhizosphere (Hemelda and Noutoshi, 2025; Wankhade et al., 2025). Meanwhile, plants utilize pattern-triggered immunity (PTI) and effector-triggered immunity (ETI) to detect microbial signals and to control pathogen invasion (Nguyen et al., 2021). They can increase the acquisition of nutrients, lead to the production of antimicrobial metabolites, siderophores, lytic enzymes, volatile organic compounds (VOCs), and quorum-quenching molecules, and induce systemic resistance (ISR), thus fortifying plant defense and resilience (Timofeeva et al., 2023; Vacheron et al., 2013; Yu et al., 2019, Yu et al., 2022).

In contrast, pathogens use secretion systems (Type I-VI), virulence effectors, phytotoxins, cell wall-degrading enzymes, and molecular mimicry to inhibit plant immunity, alter host metabolism and disrupt beneficial microbial colonization (Matte et al., 2024; Ramachandran et al., 2021; Zhang et al., 2022). They also have the capacity to alter the exudation of roots and the existing microbial communities, creating an environment that is conducive to infection (Jin et al., 2024). At the same time, the interactions between beneficial and pathogenic microorganisms on the ecological niche and/or on the limiting resource is influenced by their ability to form biofilm, produce antimicrobial metabolites, use siderophores, and by chemical communication, while additional environmental factors like soil type, pH, moisture, nutrient availability, and abiotic stress can impact the outcome of these interactions (Abdul Rahman et al., 2021; Gu et al., 2020).

The advent of multi-omics technologies such as metagenomics, metatranscriptomics, proteomics, metabolomics, spatial imaging, and single-cell analyses has revolutionized knowledge of rhizosphere ecology and its dynamic feedback between plants, microbial communities and environmental conditions (Bhimani et al., 2024; Chaparro et al., 2014). These strategies show that the rhizosphere is a complex network of life, in which plants can select beneficial partners, pathogens develop counterstrategies, and microbial communities as a whole suppress or promote disease (Li et al., 2021). This information unlocks new possibilities for microbiome engineering, synthetic microbial consortia, targeted biocontrol, and disease-suppressive soils to enhance sustainable and climate-resilient agriculture (Moretti et al., 2024; Pandey and Saharan, 2025).

While several reviews have covered immunity in plants, disease-suppressive microbiomes, or plant-microbe interaction in isolation, there is little integration of plants with beneficial microbes and pathogens that underpins the mechanistic response (Gupta et al., 2021; Mashabela et al., 2022). This review addresses the following points: (i) summarizing the molecular mechanisms responsible for plant-beneficial microbe-pathogen interactions, (ii) describing the role of plant immunity, root exudates, root microbe competition and metabolite-mediated signaling in shaping the rhizosphere, (iii) outlining recent advances in multi-omics technologies, (iv) discussing opportunities for microbiome engineering and synthetic microbial consortia, and (v) pinpointing future research priorities for sustainable crop protection by using the plant and soil microbiome.

## 2 The battle in the rhizosphere as a dynamic interaction

The rhizosphere is a dynamic environment that hosts a complex mix of physical, chemical, and ecological interactions among plants, beneficial microbes, and pathogens, all of which influence plant health and disease outcomes (Ling et al., 2022). The microbial recruitment, colonization and activity in this narrow soil zone are controlled by root exudates such as sugars, amino acids, organic acids, phenolics, flavonoids and signaling molecules (da Silva Lima et al., 2014; Upadhyay et al., 2026). These exudates provide nutrients and chemical signals, which in turn can favor or prevent the proliferation of chemical signal favors or prevents the proliferation of beneficial microbes or pathogens and influence the assembly of the rhizosphere microbiome (Philippot et al., 2013; Upadhyay, 2026a, Upadhyay, 2026b). The rhizosphere is therefore the first line of defense in which plants actively select antagonistic fungi and plant growth-promoting rhizobacteria (PGPR) to strengthen their defense against invading pathogens (Vives-Peris et al., 2020).

Plants dynamically adjust their root exudation in response to pathogen attack to attract beneficial microbes that can produce antibiotics, siderophores, volatile organic compounds (VOCs), lytic enzymes and other bioactive metabolites that inhibit pathogen establishment. This adaptive reaction is called the “cry-for-help” strategy, which helps to promote disease suppression through enrichment of protective microbial communities (Liu et al., 2024). Similarly, plants facing aboveground herbivores benefit from recruitment of beneficial microbes that suppress herbivore damage and enhance the fitness of the plants, via an indirect defense system (Dicke and Baldwin, 2010).

On the other hand, pathogens alter the rhizosphere by exuding virulence effectors, phytotoxins, hydrolytic enzymes, and signaling molecules that weaken the plant’s defense mechanism, change the composition of root exudates, and disrupt the beneficial microbes (Upadhyay, 2026b; Yang et al., 2024). In addition, many pathogens alter the availability of carbon and iron to improve their competitive ability and to disrupt microbial quorum sensing (Rovenich et al., 2014). This means that ecological niche, nutrient and iron competition, as well as production of antimicrobial metabolites and disruption of signaling pathways, play a major role in the establishment of pathogens and/or disease suppression (Berendsen et al., 2012; Du et al., 2025).

Soil physicochemical properties like pH, moisture, temperature, organic matter and agricultural management practices play a crucial role in determining the outcome of this ecological competition (Abdul Rahman et al., 2021). Recent developments in multi-omics technologies such as metagenomics, metatranscriptomics, metabolomics and imaging have made the rhizosphere a more complex biochemical network with coordinated signaling, metabolic exchange and microbial interactions (Rajguru et al., 2024). The comprehension of these mechanisms opens up the doors for microbiome engineering, developing synthetic microbial consortia, and targeted biocontrol strategies to improve disease suppression and sustainable agriculture (Dubey et al., 2025; Sahoo et al., 2025).

### 2.1 Physical and chemical landscape of the rhizosphere

The rhizosphere is a dynamic microenvironment influenced by root architecture, soil physicochemical properties and root-microbe interaction (Fadiji et al., 2025). The physical and chemical properties control microbial recruitment, nutrient availability and may influence the outcome of interactions between plant and beneficial microbes and pathogens (Pantigoso et al., 2022). The integrated physical and chemical landscape of the rhizosphere and its effect on plant-microbe interaction is shown in **Figure 1**. While soil moisture, pH, temperature, gaseous exchange, and redox conditions affect microbial activity and nutrient transformations, root exudates play a role as central regulators, affecting microbial recruitment, nutrient availability and metabolic communication. All these physicochemical drivers contribute to shaping microbial community assembly, competition, and biochemical signals, which ultimately affect nutrient uptake, plant immunity, disease suppression, and the overall functionality of the rhizosphere.

**Figure 1:**
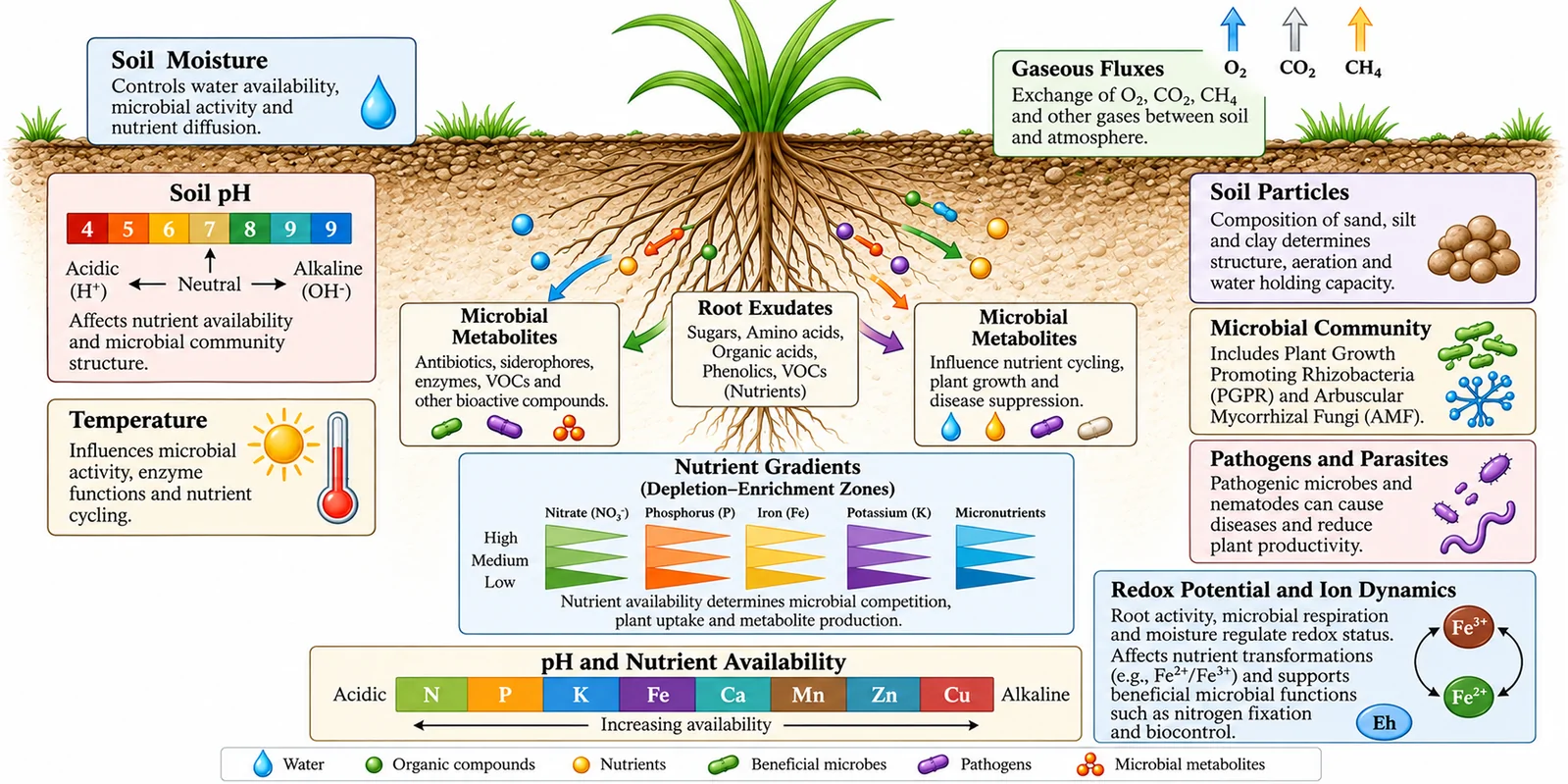
The physical and chemical architecture of the rhizosphere and how it influences plant-beneficial microbe-pathogen interactions. This figure represents a schematic description of the main physical and chemical factors that control the rhizosphere microenvironment. The interactions of root exudates, soil moisture, pH, temperature, gaseous exchange, soil particles, nutrient gradients, microbial metabolites, redox potential, and microbial communities all affect nutrient availability, microbial recruitment, metabolic activity, and plant-beneficial microbe-pathogen interactions. The processes are integrated and responsible for rhizosphere function, disease suppression and plant productivity.

#### 2.1.1 Physical structure of the rhizosphere

##### 2.1.1.1 Soil aggregation and microstructure

At all times, plant roots change the soil architecture by creating aggregates, binding soil particles and modifying the pore network (Hamza et al., 2025). The polysaccharide-rich mucilage secreted by the roots promotes soil cohesion, water retention, and formation of microhabitats (Li et al., 2024). These aggregates create microsites with different moisture, oxygen and organic carbon levels and thus create a greater diversity of niches for microbes (Khabir et al., 2023).

##### 2.1.1.2 Water and oxygen gradients

The root water uptake and respiration generate strong moisture gradients and oxygen gradients, which strongly affect the distribution of microorganisms (Tan et al., 2021). The rhizoplane is dominated by aerobic microorganisms, and oxygen-limited microsites by anaerobic and microaerophilic microorganisms (Bhattacharyya et al., 2023). Water depletion zones can also control nutrient diffusion and the mobility of chemicals in the rhizosphere (York et al., 2016).

##### 2.1.1.3 Root surface topography

Root hairs, border cells and exudate coatings generate unique micro-habitats that help microbial attachment and colonization (Omelyanchuk et al., 2024). The rhizoplane is therefore typically more microbiologically active than the bulk soil due to a higher availability of attachment sites and hot spots of nutrients.

#### 2.1.2 Chemical properties of the rhizosphere

##### 2.1.2.1 Root exudates and nutrient hot spots

Sugars, amino acids, organic acids, phenolics, fatty acids, and signaling molecules are among the key chemical components of the rhizosphere, called root exudates, that dictate the rhizosphere processes (Sibanyoni et al., 2026). These compounds act as carbon sources and as signals to attract desirable microorganisms, to control the composition of microorganisms and to affect the activity of pathogens (Feng et al., 2025). They can be very dynamic and can change depending on the nutrient status of the soil, the plant growth stage, and environmental stress (de Haas et al., 2025).

##### 2.1.2.2 pH modification

Rhizosphere pH changes are actively done by plants through the process of extrusion of protons and the release of organic acids (Kanwar et al., 2025). Rhizosphere acidification may facilitate the uptake of nutrients like phosphorus and also affect the structure of microbial communities (Lurthy et al., 2021). In contrast, nitrate uptake tends to cause alkalinization, and consequently, nitrate-induced chemical gradients can occur at a distance of only a few millimeters from the root surface (Wang and Tang, 2018).

##### 2.1.2.3 Nutrient gradients (N, P, Fe, K, micronutrients)

Zones of nutrient deficiency and sufficiency develop around the root, influencing microbial activity and plant-microbe interactions (York et al., 2016). In addition to iron, gradients of nitrogen, phosphorus and other nutrients affect microbial competition, survival mechanisms and production of specialized metabolites, favoring microorganisms that have the ability to produce siderophores, which are involved in disease suppression (Gu et al., 2020).

##### 2.1.2.4 The study of redox potential and ion dynamics

The root metabolism, microbial respiration and soil water content (Husson, 2013) influence rhizosphere redox potential. These will affect the bioavailability of the nutrients, especially the Fe²^+^/Fe³^+^ ratio, as well as the activity of beneficial microbes that participate in nitrogen fixation and biological control (Molnár et al., 2023).

#### 2.1.3 Explored interactions between physical and chemical factors

The rhizosphere is a unified physicochemical system, where physical and chemical properties work together to regulate the assembly of microbial communities and the functioning of the ecosystem as a whole, and not separately (Chaparro et al., 2014; Upadhyay, 2026b). To enhance nutrient-use efficiency and develop strategies for sustainable agriculture based on the microbiome, understanding these interactions is crucial (Griffin et al., 2024). Root mucilage, for instance, regulates water distribution and nutrient diffusion; soil aggregation affects oxygen supply and microbial metabolism; root-induced pH gradients affect enzyme activity, carbon turnover and nutrient transformations (Oburger et al., 2022). These factors collectively influence resource availability, niche differentiation of microorganisms, and signaling networks which regulate interactions between plant beneficial microbes and pathogens (Philippot et al., 2013). Soils are therefore strongly affected by soil structure, moisture, oxygen, pH and gradients of nutrients, which all have an impact on microbial colonization, metabolic activity, plant performance, and disease suppression (Hinsinger et al., 2009; Philippot et al., 2013).

##### 2.1.3.1 Nutrient acquisition

The chemical and physical environment around the rhizosphere can affect the transformation, movement, and bioavailability of nutrients (Liu et al., 2022). Beneficial microbes improve nutrient acquisition by biological nitrogen fixation, phosphate solubilization, mobilization of iron and mineral weathering, while pathogens adversely affect nutrient acquisition by damaging root tissue and other nutrient fluxes (Richardson et al., 2009; Timofeeva et al., 2023). Microbiological recruitment, nutrient cycling and competitive interactions in the rhizosphere are additionally regulated by root-induced changes in pH and exudate composition (Feng et al., 2024; Philippot et al., 2013).

##### 2.1.3.2 Photosynthetic efficiency

The effects of rhizosphere processes on photosynthetic performance are indirect through control of nutrient availability and plant stress responses. Beneficial microbes stimulate the biosynthesis of chlorophyll, increase the stability of PSII, increase the uptake of nutrients, and regulate the hormones and metabolism in the plant to reduce oxidative stress (Olanrewaju et al., 2017). In contrast, pathogen infection causes a reduction in photosynthetic efficiency through the activation of reactive oxygen species (ROS), vascular dysfunction and stomatal impairment (Berger et al., 2007). Thus, water and nutrient availability (regulated by the rhizosphere) directly impact carbon assimilation and plant productivity (Vurukonda et al., 2016).

##### 2.1.3.3 Carbon allocation

Carbon allocation is related to ecological processes in the rhizosphere and metabolism of plants. A significant portion of the photosynthetically fixed carbon is also released as root exudates that can be used to guide the assembly of the microbial community and to selectively attract beneficial microbes (Sasse et al., 2018). The balance of carbon partitioning for growth, defense, and rhizosphere investment is continually shifted by microbial colonization, pathogen attack, and availability of nutrients. Carbon reallocated to defense pathways is lost from other processes, including belowground carbon allocation, which is accompanied by an increase in nutrient uptake and stress tolerance by beneficial microbes (Berendsen et al., 2012).

##### 2.1.3.4 Root system architecture

The root architecture is the result of the interactions between physicochemical conditions and microbial signaling. Beneficial microbes induce phytohormone production, volatile compounds and/or signaling molecules that promote root elongation, lateral root emergence and root hair extension, which enhance resource acquisition and soil exploration (Lugtenberg and Kamilova, 2009; Vacheron et al., 2013; Upadhyay, 2026c). On the other hand, pathogens have a negative effect on root growth due to direct damage to tissues and interference with hormones, microbial habitat changes and rhizosphere interaction changes (Berendsen et al., 2012; Pieterse et al., 2014).

##### 2.1.3.5 Water-use efficiency

The water-use efficiency is a result of the interaction and coordination between soil, roots, and microorganisms. Microbial extracellular polymers, mucilage, pore connectivity and soil aggregation control water retention and make it available to the rhizosphere (Batista et al., 2024; Costa et al., 2018; Vurukonda et al., 2016). Beneficial microbes enhance soil aggregation by producing exopolysaccharides, improve root hydraulic conductivity, osmotic adjustment and signaling in response to water stress (Costa et al., 2018; Kumar et al., 2024; Timmusk et al., 2014; Vurukonda et al., 2016). Pathogenic microorganisms, on the other hand, cause damage to the vascular tissues, leading to a decrease in the uptake of water and an increase in drought susceptibility. Therefore, the combined action of physicochemical and biological processes in the rhizosphere is essential for efficient water use (Philippot et al., 2013; Vurukonda et al., 2016).

Together, the physical and chemical characteristics of the rhizosphere control resource availability, the metabolism of microorganisms, communication between microorganisms and plants, and competition between microorganisms and pathogens, and thus influence plant-beneficial microbe-pathogen interactions (Berendsen et al., 2012; Philippot et al., 2013; Zhuang et al., 2024). These integrated processes govern microbiome assembly, plant health, disease suppression and crop resilience in the face of fluctuating environmental conditions, and lay the foundation for microbiome-based sustainable agriculture (Dubey et al., 2025; Trivedi et al., 2020).

## 3 Plant strategies in triangular warfare

Plants use a range of integrated defense mechanisms, including complex hormonal crosstalk (e.g. salicylic acid, jasmonic acid and ethylene), which fine-tunes defensive signaling networks, as well as sophisticated innate immune responses including PTI and ETI and root-mediated recruitment and modulation of beneficial microbes that collectively shape plant resistance and resilience against pathogens while balancing growth and stress responses (Pandey et al., 2025). Plants use various interconnected strategies such as selective root exudation, immune priming, rhizosphere chemical modulation and recruitment of beneficial microbes to build a resilient rhizosphere microbiome that is resistant to invasion by pathogens and to maintain a healthy plant state (**Figure 2**).

**Figure 2:**
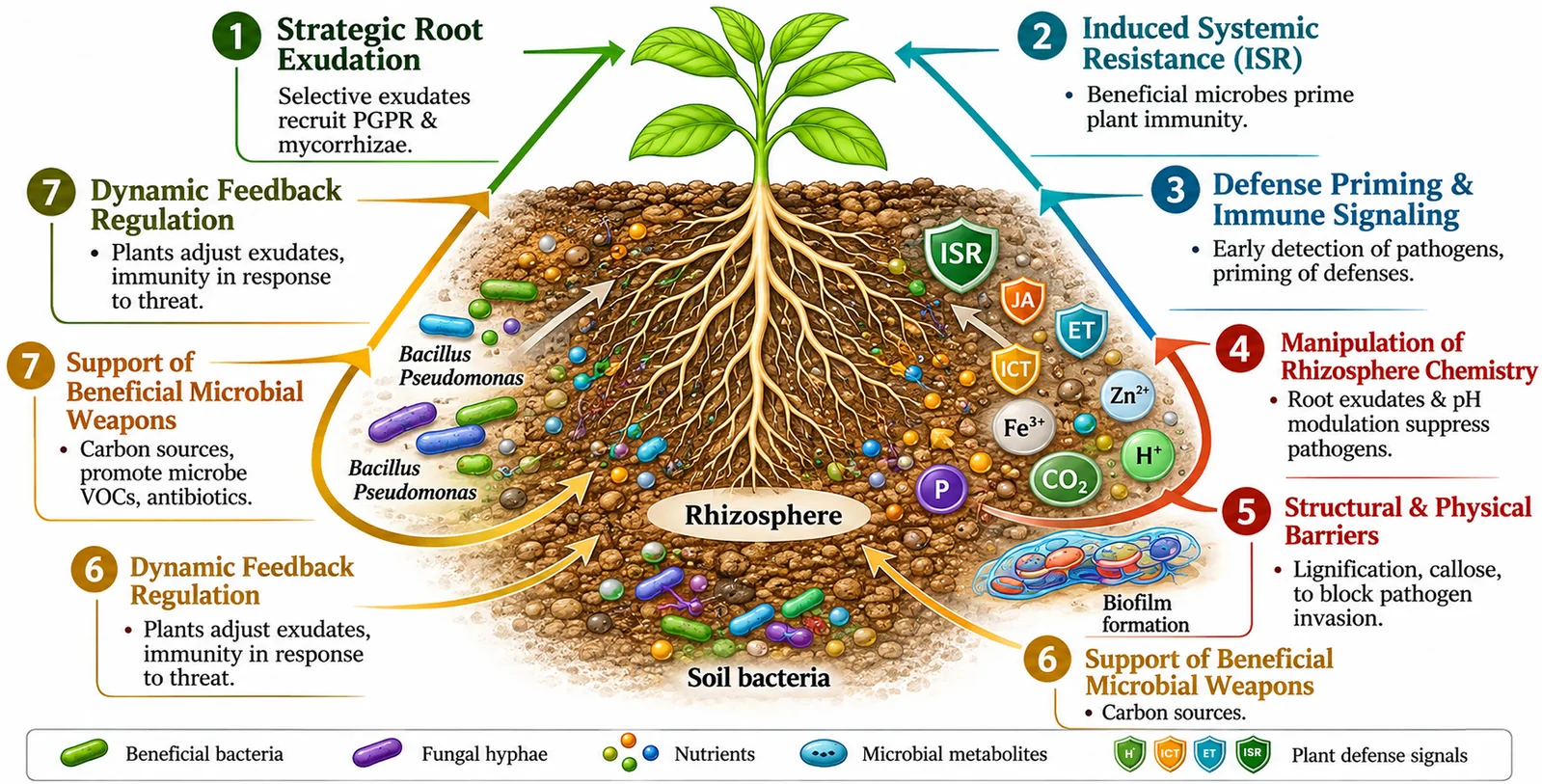
Rhizosphere integrated signaling network regulating plant-beneficial interaction of microbe-pathogen. This conceptual diagram shows integrated defense strategies used by plants to control the microbial community in the rhizosphere. Positive recruitment of beneficial microbes by selective root exudation, activation of systemic resistance (ISR) and defense priming for higher immunity. Plants, at the same time, alter the rhizosphere chemistry, strengthen structural barriers, and promote the production of beneficial microbes which produce antimicrobial compounds, volatile organic compounds (VOCs), lytic enzymes and biofilms that inhibit the establishment of pathogens. The interplay among root exudation, microbial activity and plant immune signaling is dynamic and continually governs rhizosphere homeostasis, leading to disease suppression, nutrient acquisition, and sustainable plant health.

### 3.1 Plant innate immunity

Plant innate immunity (de Wit, 2007) is the natural defense mechanism in which plants recognize and react to the invasion of pathogens, including bacteria, fungi, viruses, nematodes and insect herbivores. Unlike animals that possess an adaptive immune system, plants are unable to rely on adaptive immune processes for defense, and therefore are dependent on innate immune processes (Spoel and Dong, 2012). Pattern-triggered immunity (PTI), which is triggered by cell-surface pattern recognition receptors (PRRs), and ETI, which is mediated by intracellular nucleotide-binding leucine-rich repeat (NLR) proteins that identify pathogen effectors, make up this multilayered surveillance system (Nabi et al., 2024). To check pathogen growth and spread, these immune layers act synergically to trigger downstream signaling pathways that involve influx of calcium, ROS, mitogen-activated protein kinase (MAPK) cascades, phytohormones and defensive gene expression (Dodds et al., 2024).

#### 3.1.1 Pattern triggered immunity

The first level of innate immunity in plants, called PTI, is activated by the recognition of conserved microbial entities called pathogen- or microbe-associated molecular patterns (PAMPs/MAMPs) (Li et al., 2016). Plant cells have PRRs on the surface that recognize these molecular patterns, such as bacterial flagellin, fungal chitin, elongation factor Tu (EF-Tu), and lipopolysaccharides (Trdá et al., 2015). Once the PAMPs are recognized by PRRs like FLS2, EFR and CERK1, a signaling pathway is activated (Macho and Zipfel, 2014). This cascade leads to a swift influx of calcium (Ca^2+^) into the cell, formation of reactive oxygen species (ROS), activation of mitogen-activated protein kinase (MAPK) pathways, and a wide transcriptomic reprogramming (Köster et al., 2022). The early responses lead to outputs of defense mechanisms, which collectively reduce the ability of the pathogen to colonize the plant, such as production of antimicrobial compounds, induction of pathogenesis-related (PR) genes, and reinforcement of the cell wall (callose deposition) (Wu et al., 2014a). In connection with its ability to provide broad-spectrum and long-lasting resistance and its role as an important defense barrier that pathogens need to breach through effector secretion, PTI is tied to ETI (Yu et al., 2024).

#### 3.1.2 Effector-triggered immunity

The second layer of plant innate immunity is called effector-triggered immunity (ETI), and it works as a powerful intracellular defense mechanism to support PTI, the first layer (Nguyen et al., 2021). ETI is activated by the detection of pathogen-derived effector proteins by the intracellular receptors that contain nucleotide-binding leucine-rich repeats (NLRs), with the pathogens providing the effector proteins to suppress PTI and facilitate infection. Unlike PTI, which provides general defense against pathogens, ETI is characterized by highly specific defense responses, such as transcriptional reprogramming, production of reactive oxygen species (ROS), influx of calcium and activation of mitogen-activated protein kinase (MAPK) cascades, and these responses are generally strong and often persistent (Nguyen et al., 2021). One of the characteristics of ETI is hypersensitive response (HR), also known as localized programmed cell death at the infection site, due to which pathogen growth is restricted (Wu et al., 2014b). Additionally, ETI increases defense pathways that may have been inhibited by pathogens and effectors and fortifies systemic acquired resistance (SAR) (Ding et al., 2022). Recent studies have demonstrated that the two are not completely independent, with both having downstream-signaling components in common and PTI and ETI amplifying each other’s responses (Yuan et al., 2021). ETI allows plants to develop a comprehensive multi-layer defense system that provides an effective barrier to pathogen penetration without adversely affecting other stress factors and growth (Islam et al., 2024).

#### 3.1.3 Hormonal crosstalk in defense during rhizosphere triangular warfare

The rhizosphere harbors a complex environment of beneficial microbes, pathogens and plant roots, with their interactions being dynamic and competitive (Chepsergon and Moleleki, 2023). Such interactions between phytohormones constitute an important regulatory node in this tripartite warfare scenario, enabling plants to reduce fitness costs whilst interpreting multiple microbial signals and initiating appropriate defense strategies (Pieterse et al., 2012). Plant hormones do not act alone, but rather in concert to affect trade-offs in growth stress, recruitment of microbes, and activation of immunity (Pieterse et al., 2012).

##### 3.1.3.1 Core defense hormones: salicylic acid, jasmonic acid, and ethylene

The main regulator of resistance to biotrophic and hemibiotrophic diseases, such as several bacteria and fungi that infect roots, is salicylic acid (SA) (Denancé et al., 2013). The SAR is built up, and pathogenesis-related (PR) genes are transcriptionally activated when SA accumulates, resulting in sustained plant defense (Zheng and Xinnian, 2013). The stimulation of “good” microorganisms associated with disease suppression by SA signaling also indirectly impacts the composition of the microbial community because of changes in rhizosphere exudation patterns (Lebeis et al., 2015). In addition to its extensive role in abiotic stress tolerance, salicylic acid (SA), also known as salicylate, is ubiquitous in terrestrial plants and has lately gained recognition for its role in defense mechanisms and plant responses to pathogen stressors (Chhana, 2025). The pathogen-specific, interconnected, and overlapping crucial role of SA in plant defense and immunity has been reported in several studies thus far (Ullah et al., 2023). According to Ullah et al. (2023), salicylic acid is principally in charge of triggering defense reactions against biotrophic and hemibiotrophic infections.

The ethylene (ET) and jasmonic acid (JA) pathways are mostly triggered by herbivorous insects and necrotrophic diseases (Pieterse et al., 2012). JA and ET induce the expression of defense genes that encode antimicrobial peptides, secondary metabolites and enzymes that strengthen the cell wall (Van der Does et al., 2013). Of particular note, many beneficial rhizosphere microbes can induce a primed state of defense (ISR) through the JA/ET pathway, which allows for rapid and strong responses when attacked by pathogens without activation of a basal defense (van Loon et al., 1998). To defend plants against infections, SA and JA phytohormones work both antagonistically and cooperatively in a complex web of signaling pathways (Ali et al., 2024b).

##### 3.1.3.2 Hormonal antagonism and synergy in rhizosphere

The antagonistic interaction between the SA and JA pathways, which enables plants to adapt defenses to the lifestyle of pathogens, is a characteristic of hormonal crosstalk (Pieterse et al., 2012). By triggering JA signaling to inhibit SA-mediated defenses or vice versa, numerous soil-borne pathogens try to control this antagonistic relationship (Zhang et al., 2025). By adjusting hormone signaling, helpful bacteria resist this tactic and guarantee efficient defense activation without jeopardizing beneficial plant-microbe relationships (Zhu et al., 2022). Furthermore, there are synergistic interactions between JA and ET, such interactions being effective in controlling root-associated defensive responses and ISR (Shoresh et al., 2005). When no pathogen is a threat, this hormonal control ensures that defense is activated in a time- and space-limited fashion, this way preventing unnecessary energy loss (Vos et al., 2015).

### 3.2 Integrated signaling network governing rhizosphere triangular warfare

Plants use an integrated signaling network to identify and discriminate beneficial rhizosphere microbes from invading pathogens and initiate appropriate defense responses (Pieterse et al., 2014). The first line of defense is PTI, which is activated by the recognition of conserved microbial molecules by pattern recognition receptors (PRRs) (Zipfel, 2014). PTI triggers rapid Ca²^+^ influx, production and accumulation of reactive oxygen species (ROS), activation of mitogen-activated protein kinase (MAPK) cascades and activation of WRKY transcription factors, which results in the induction of defense-related genes. Pathogens use effector proteins to enter host cells and block immune signaling as a strategy to overcome PTI (Bigeard et al., 2015; Xiangzong and Shuqun, 2013). The intracellular nucleotide-binding leucine-rich repeat (NLR) receptors detect these effectors and trigger ETI, which is a more powerful and persistent defense with enhanced ROS accumulation, MAPK signaling and localized immune responses (Jones and Dangl, 2006).

These immune pathways are further interconnected by hormonal signaling and help mediate pathogen resistance while maintaining healthy interactions with microorganisms. Nonexpresser of pathogenesis-related genes 1 (NPR1) is the central regulator of SA-mediated signaling and systemic acquired resistance (SAR) (Zheng and Xinnian, 2013; Pieterse et al., 2012; Wasternack and Song, 2017), while MYC2 coordinates jasmonic acid (JA)-dependent signaling associated with ISR (Pieterse et al., 2012; Wasternack and Song, 2017). Further, ethylene-responsive transcription factor EIN3 interacts with JA signaling to boost defense responses without compromising rhizosphere interactions. When combined, these interconnected signaling pathways coordinate microbial recognition, immune activation and hormonal control, allowing plants to control pathogens while maintaining the rhizosphere beneficial microbial flora (Broekgaarden et al., 2015; Pieterse et al., 2014) (**Figure 3**).

**Figure 3:**
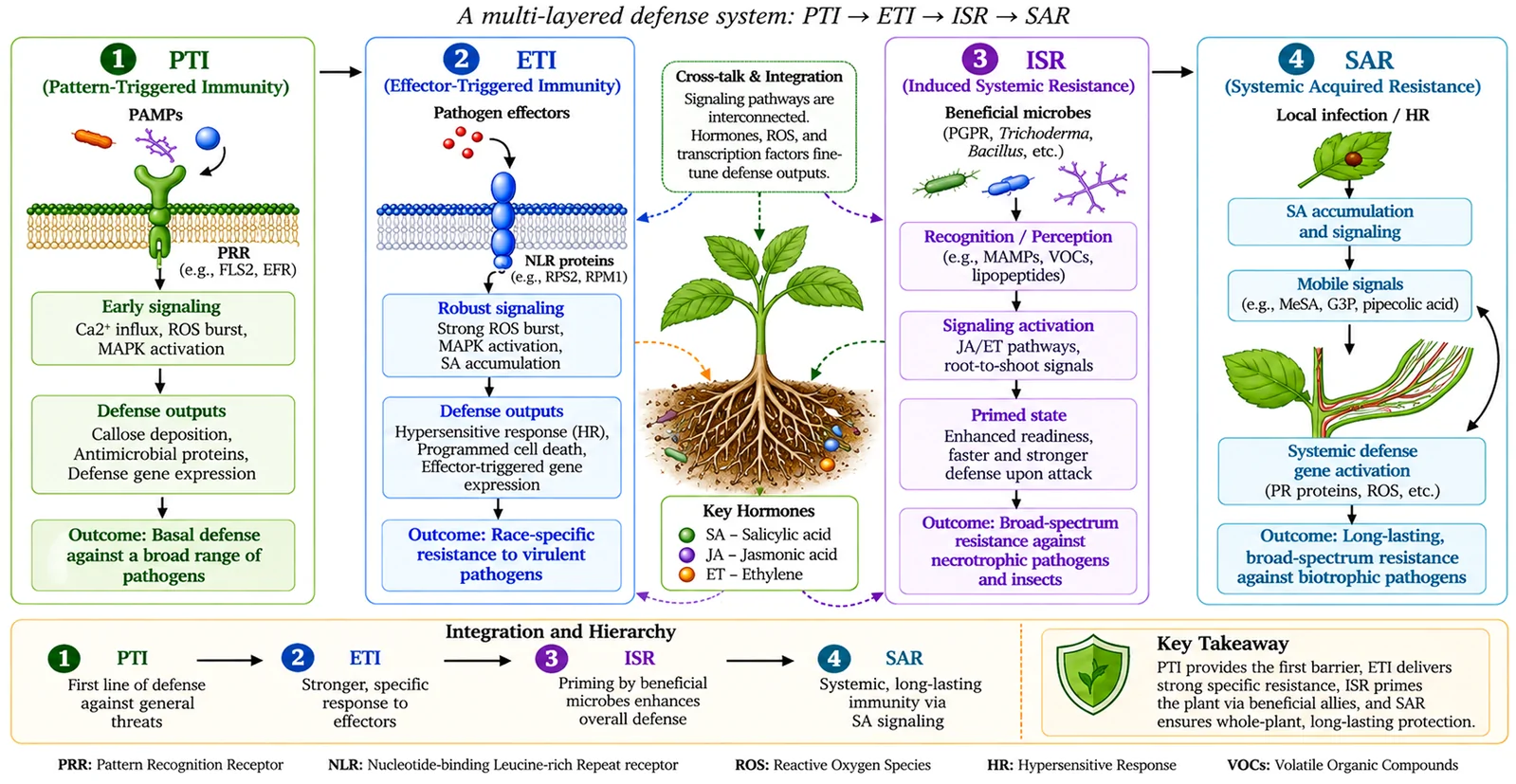
Integrated signaling network regulating plant-beneficial microbe-pathogen interactions in the rhizosphere. The network depicts the coordinated interaction of plant immune signaling pathways controlled by salicylic acid (SA), jasmonic acid (JA), ethylene (ET), and reactive oxygen species (ROS), such as pattern-triggered immunity (PTI), effector-triggered immunity (ETI), induced systemic resistance (ISR), and systemic acquired resistance (SAR). Beneficial microorganisms modify these pathways to promote plant growth, nutrient uptake, and disease resistance, whereas pathogens use virulence mechanisms to inhibit or evade host protection. Cross-talk between these signaling pathways integrates microbial sensing, root exudates, hormonal responses, and defense-related gene expression to influence the outcome of plant-microbe interactions and maintain rhizosphere health.

## 4 Molecular mechanisms leading to triangular interactions (plant-beneficial microbe–pathogen)

The rhizosphere is a constantly changing molecular interface in which plants, beneficial microbes, and pathogens interact in complex ways, with their interconnected signaling processes playing a key role in plant health, plant microbiome assembly and disease outcome (Yang et al., 2024). For plants to distinguish between beneficial microbes and pathogens, they have innate immune system mechanisms that involve pattern recognition receptors (PRRs) triggering PTI and intracellular nucleotide-binding leucine-rich repeat (NLR) receptors detecting pathogen effectors to initiate ETI (Nabi et al., 2024). Salicylic acid (SA), JA, and ET signaling further regulate this immune specificity by coordinating defense responses and ensuring beneficial microbial association (Yang et al., 2015). In parallel, plants influence rhizosphere microbiome structure by their dynamic exudation of primary and secondary metabolites that serve as nutrient sources and signaling molecules to attract beneficial microbes, especially under stress (Wankhade et al., 2025). Beneficial microbes subsequently boost the plant’s fitness by synthesizing antimicrobial metabolites, volatile organic compounds, siderophores, phytohormones and immune-modulating compounds that reduce pathogen virulence and lead to systemic resistance (ISR) at low fitness costs (Pieterse et al., 2014). In addition to these plant-mediated processes, there is a synergistic molecular network in the rhizosphere, involving direct microbial competition through antibiosis, nutrient sequestration, and niche exclusion that helps maintain rhizosphere homeostasis and reduce the development of diseases (Jamil et al., 2022) (**Table 1**).

**Table 1:** Molecular mechanisms driving plant-beneficial microbe-pathogen interactions.

Mechanism	Description	Role in tripartite interaction (plant-beneficial microbe-pathogen)	References
Root exudate signaling	Plants release root exudates (e.g., amino acids, sugars, organic acids) that act as chemical signals in the rhizosphere.	Attracts beneficial microbes (PGPR/PGPF) and can shape microbial community composition; it also influences pathogen behavior.	Yang et al., 2024
Microbial chemotaxis and attachment	Beneficial microbes detect root exudates *via* chemoreceptors and migrate toward roots; attach using adhesins and exopolysaccharides.	Enables beneficial microbes to colonize niches and compete with pathogens for space and resources.	Yang et al., 2024
Pattern-triggered immunity (PTI)	Plant PRRs (Pattern recognition receptors) recognize microbial patterns (PAMPs/MAMPs) to trigger basal immune signaling.	First line of plant defense; can be activated by pathogens and also modulated by beneficial microbes to “prime” defenses.	Yang et al., 2024
Effector-triggered immunity (ETI)	Pathogen effectors are recognized by plant R-proteins, leading to robust immune responses.	Pathogens use effectors to suppress defenses, while beneficial microbes can indirectly enhance host alertness.	Liu et al., 2025
Induced systemic resistance (ISR)	Beneficial microbes (PGPR/PGPF) trigger systemic defense via JA and ET signaling pathways.	Enhances plant resistance against pathogens without direct pathogen exposure, critical in tripartite defense.	Yu et al., 2022
Quorum sensing (QS)	Microbial communication via diffusible signals (e.g., AHLs) affects gene regulation, biofilm formation, and virulence.	Beneficial microbes use QS for root colonization/biofilms; pathogens use QS to coordinate infection. Competitive QS outcomes shape interactions.	Farrar et al., 2014
Secondary metabolites/VOCs	Beneficial microbes release volatile organic compounds (VOCs) and secondary metabolites (e.g., siderophores) that influence plant signaling and microbial competition.	VOCs can induce ISR and suppress pathogen growth, enhancing plant resilience.	Jain et al., 2024
Hormonal crosstalk modulation	Interactions involve phytohormones (auxins, cytokinins, SA, JA, ET), which are produced/modified by microorganisms and perceived by plants.	Beneficial microbes manipulate hormonal balance to stimulate growth/defense; pathogens manipulate hormones to suppress immunity.	Liu et al., 2025
Biofilm formation and competition for niches	Beneficial microbes form biofilms on roots that block pathogen colonization and provide physical/chemical defense.	Biofilms enhance survival/colonization of beneficial microbes, reducing pathogen establishment.	Farrar et al., 2014
Metabolome reshaping and chemical dialogues	Complex chemical exchanges (metabolites/signals) define recruitment, suppression, and activation of defense and growth pathways.	Metabolomic profiling reveals how tripartite signals influence plant defense prioritization.	Sibanyoni et al., 2026

AHLs, N-Acyl homoserine lactones; PGPR, Plant Growth Promoting Rhizobacteria; PGPF, Plant Growth Promoting Fungi; PAMPs, Pathogen-Associated Molecular Patterns; MAMPs, Microbe-Associated Molecular Patterns; SA, Salicylic Acid.

### 4.1 Secretion systems

The microbial secretion systems play an important role in plant-beneficial microbe-pathogen interactions through virulence regulation, microbial competition, and host colonization (Trivedi et al., 2020; Upadhyay, 2026d). A few of these, including the Type III (T3SS), Type IV (T4SS) and Type VI (T6SS) secretion systems, are critical in determining rhizosphere ecology (Green and Mecsas, 2016).

The T3SS is mainly associated with Gram-negative phytopathogens, which secrete effector proteins directly into plant cells, resulting in a suppression of PTI and promoting the infection process (Galán and Waksman, 2018). Plants do have specific intracellular immune receptors, however, that detect effectors and trigger ETI, thereby preventing pathogens from colonizing the plant (Cui et al., 2015).

The T4SS is active in pathogenic and mutualistic microorganisms, and is responsible for the delivery of proteins or nucleic acids into either plant cells or microbial cells. Pathogenic bacteria use T4SS to increase their virulence, but positive microorganisms use it to facilitate horizontal gene transfer, biofilm formation and stable association with plant roots, thus promoting long-term plant-microbe relations (Costa et al., 2024).

The T6SS is mainly responsible for intermicrobial competition by injecting antibacterial effectors into the cells of the neighboring microorganisms, thereby eliminating competitors and increasing the ecological fitness of the T6SS producer (Russell et al., 2014). Rhizobacteria are often able to use T6SS for pathogen protection, thereby creating disease-suppressive microbial communities around plant roots.

In addition to their function in pathogenicity, secretion systems are also important in beneficial plant-microbe communication. The plant growth-promoting rhizosphere bacteria produce protein effectors and/or signaling molecules that regulate plant immunity, activate ISR, improve nutrient uptake, and colonize the roots without causing disease (Trivedi et al., 2020). Together, T3SS, T4SS and T6SS act as multi-functional molecular platforms that communicate microbial interaction, immune modulation and rhizosphere communication, which in turn dictate the stability of the microbiome and the resilience of the ecosystem (Costa et al., 2024).

### 4.2 Multi-omics perspectives

The recent developments of multi-omics technologies have revolutionized our understanding of plant-beneficial microbe–pathogen interactions, facilitating a system-wide analysis of the processes occurring in the rhizosphere (Kumar et al., 2023; Upadhyay et al., 2026). Genomics and metagenomics provide insights into the diversity of microorganisms and functional genes linked to nutrient uptake, secondary metabolite synthesis, and pathogen resistance (Chukwuneme et al., 2021). Transcriptomics studies the changes in plant and microbial gene expression that occur in response to their changing environments; during beneficial and pathogenic interactions, it provides information on how plants respond to microbial colonization, as well as microbial strategies for colonization (Roy and Solomon Maina, 2024). In addition to transcriptomics, proteomics is used to detect secreted effectors, enzymes and signaling proteins that regulate immune responses and competition with microorganisms, while metabolomics is used to decipher the chemistry of dialogue between roots, microorganisms and microbial metabolites in the form of signaling molecules (Mhlongo et al., 2018; Sahoo et al., 2025; Secomandi et al., 2025). A combination of these complementary omics approaches can help in reconstructing the rhizosphere triangle regulatory network between plant immunity, microbial activity, and environmental cues, thereby delivering a comprehensive understanding of rhizosphere triangle interactions and microbiome-based approaches for sustainable crop protection (Roy and Solomon Maina, 2024; Kumar et al., 2023).

### 4.3 Metagenomics for community profiling

By providing culture-independent characterization of the rhizosphere microbiomes, metagenomics has emerged as a key strategy for investigating microbe-pathogen interactions in plant–beneficial contexts (Fadiji et al., 2021). The Amplicon sequencing method of 16S rRNA and ITS regions can generate high-resolution taxonomic profiles that can explain the relationship between the genotype of plants and developmental stage on the one hand and the microbial community composition on the other, depending on the environmental conditions (Quiza et al., 2023). Shotgun metagenomics, on the other hand, reveals functional genes involved in nutrient cycling, secondary metabolite biosynthesis, quorum sensing, antibiotic production, and virulence, thereby giving a better understanding of the microbial ecological functions (Akinola et al., 2021). Comparative metagenomic analysis also shows that disease suppression can be achieved by increased functional abundance of microbial guilds and that microbial signatures linked with pathogen exclusion and beneficial microbial recruitment can be identified (Tracanna et al., 2021; Xiao et al., 2024). Therefore, metagenomics provides a strong platform for the understanding of the assembly of the microbiome and its role in rhizosphere health and disease suppression (Rajguru et al., 2024).

### 4.4 Transcriptomics for changes in gene expression of plants and microorganisms

Transcriptomics offers dynamic insights into gene expression changes associated with the interactions between plants and beneficial microbes and pathogens as they colonize, compete and infect the host plant (Carvalhais et al., 2012; Nobori et al., 2022). The coordinated regulation of PTI, ETI, hormone signaling and secondary metabolism in response to beneficial microbes and pathogens (Wang et al., 2020) has been discovered by plant transcriptome analyses. In general, beneficial microbes create a transcriptionally “primed” state that results in a rapid response to defense activation with low fitness costs (Pieterse et al., 2014). On the other hand, pathogen transcriptomes indicate virulence-related pathways, while microbial transcriptomes reveal genes related to root colonization, production of antimicrobial metabolites and communication with the host (Du et al., 2022). Moreover, the plant and microbial dual RNA sequencing enables the simultaneous profiling of both plant and microbial transcripts, which is unprecedented and provides novel insights into cross-kingdom signaling and coordinated regulatory networks that dictate disease resistance and rhizosphere function (Jain et al., 2024; Zhang et al., 2019).

### 4.5 Metabolomics for root exudate and metabolite mapping

Metabolomics is used to characterize metabolites excreted from plant roots and related microorganisms and to gain a comprehensive insight into the chemical interactions of plant-beneficial microbes and pathogens (Sibanyoni et al., 2026). The ability to control which sugars, amino acids, organic acids, phenolics, flavonoids and specialized metabolites are excreted from plant roots is strongly dynamic and changes throughout microbial colonization and pathogen attack, as revealed by the metabolomics of root exudates (Ku et al., 2025). These metabolites serve as a source of nutrients and potent signaling molecules that attract beneficial bacteria, inhibit pathogenic bacteria and regulate microbial behavior (Feng et al., 2024). Furthermore, the “cry for help” hypothesis was supported by metabolomics data, in which metabolites produced during stress selectively increase the abundance of protective microbial taxa and increase antagonistic activities (Rizaludin et al., 2021). Meanwhile, microbial metabolomics can discover antibiotics, siderophores, volatile organic compounds (VOCs) and quorum-sensing molecules that mediate microbial competition and immune-priming. Metabolomics integration with metagenomics and transcriptomics consequently allows the correlation of metabolic signatures with functional genes and pathways, thus offering a systems-level view of rhizosphere communication and disease suppression (Mmotla et al., 2025; Go et al., 2024; Sibanyoni et al., 2026).

Multiple metabolites produced in the rhizosphere by the roots have key roles in regulating the assembly of the rhizosphere microbiome. Coumarins, such as scopoletin and fraxetin, selectively promote the growth of beneficial microbes and mobilize iron, as well as inhibit soil-borne pathogens (Voges et al., 2019). Flavonoids and strigolactones can promote beneficial interactions between plant growth-promoting bacteria and arbuscular mycorrhizal fungi (Hassan and Mathesius, 2012), while benzoxazinoids can restructure the microbial communities and suppress pathogens (Hu et al., 2018). Similarly, organic acids like citrate and malate promote nutrient-solubilizing microorganisms and enhance nutrient availability, including P and Fe (Jacoby et al., 2017) (**Figure 4**).

**Figure 4:**
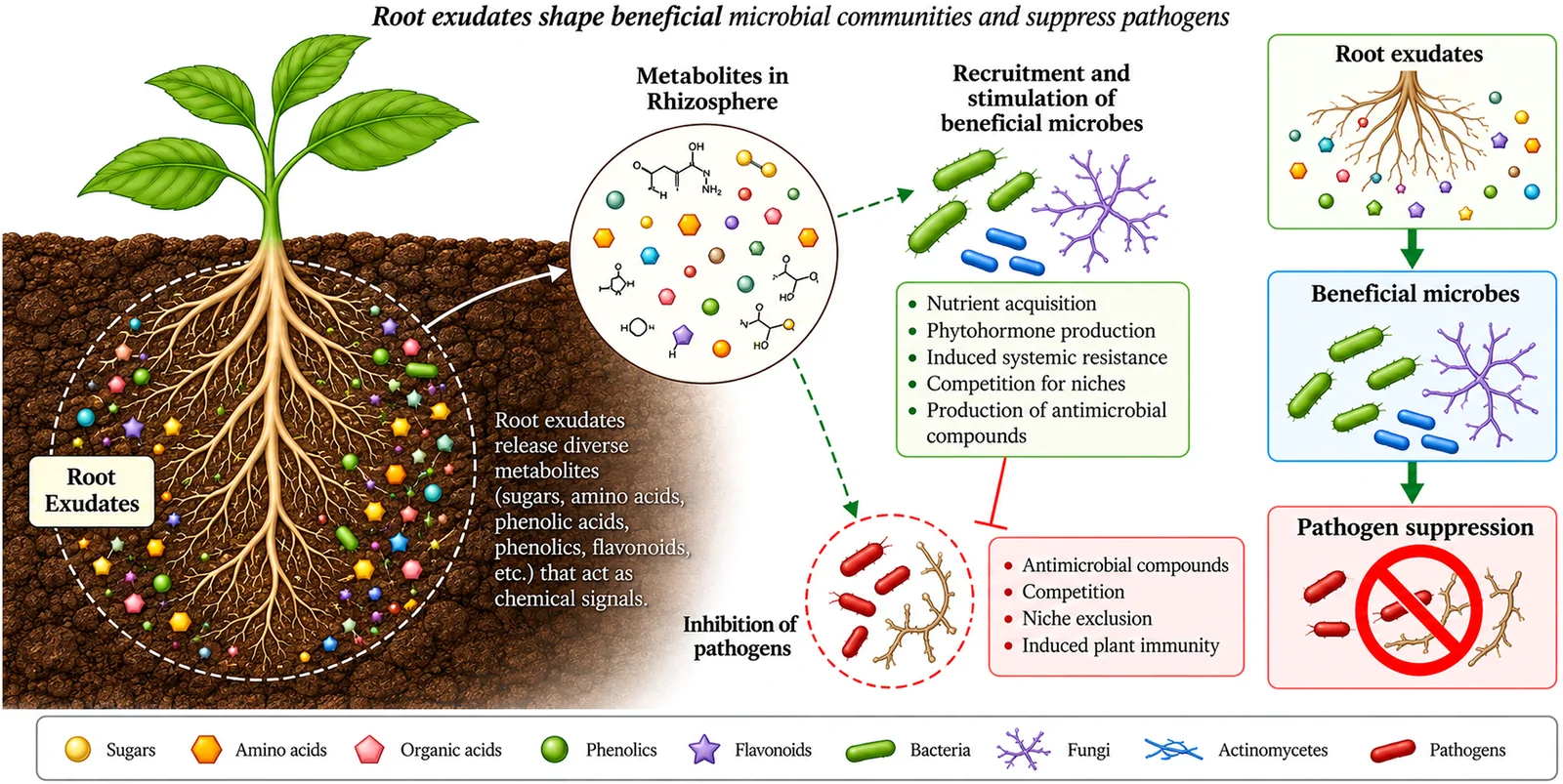
Root-to-root signaling of rhizosphere microorganisms for beneficial microbial recruitment and suppression of pathogens by metabolites. The metabolites produced by the roots (sugars, amino acids, organic acids, phenolics and flavonoids) act as a chemical cue to attract beneficial microbes and inhibit the pathogen’s establishment. These metabolite-mediated interactions help plants acquire nutrients, enhance antimicrobial activity and induce systemic resistance (ISR), as well as help in the assembly of the microbiome, thus improving rhizosphere stability and plant health.

These metabolites are secreted by specialized transporters at the level of the membrane, which tightly regulate their secretion. Altogether, these sugar efflux (SWEET) transporters, coumarin export (MATE) transporters, antimicrobial and signaling compound export (ABC) transporters and malate export (ALMT) transporters help regulate the recruitment of microorganisms and mobilization of nutrients (Chen et al., 2010; Kang et al., 2011; Voges et al., 2019). In the presence of pathogens, plants redirect the activity of transporters and exudation of metabolites to strengthen defense-related molecules and to limit molecules that are beneficial for pathogen growth, driving plants to keep rhizosphere homeostasis and favoring beneficial microbial communities (Devanna et al., 2021; Baetz and Martinoia, 2014).

### 4.6 Signaling molecules

The complexity of the interactions between the plant, beneficial microbes and pathogens in the rhizosphere is managed by chemical signaling (Ali et al., 2024a). The collective regulation of microbial communication, competition and plant defense involves antimicrobial metabolites, volatile organic compounds (VOCs) and quorum-sensing (QS) molecules (Jamil et al., 2022). In response to microbial population density, QS molecules such as N-acyl homoserine lactones and autoinducing peptides act as regulators for biofilm formation, colonization of roots, production of antibiotics, and virulence (Nachiyar et al., 2025). Pathogens use QS to increase virulence, which can be sensed or modulated by plants to reduce pathogen fitness, but beneficial microbes can use QS to enhance ecological fitness and disease suppression (Zheng et al., 2025).

The beneficial microbial VOCs are long-distance signaling molecules that promote plant growth, trigger immune reactions, and suppress the growth of pathogens without physical contact (Rani et al., 2023; Raza et al., 2021). They may also impact phytohormone signaling and inhibit pathogen sporulation and mycelial growth, while pathogen VOCs can alter plant metabolic activities to promote infection (Cordovez et al., 2017; Surovy et al., 2023). Furthermore, at sub-inhibitory levels, antimicrobial metabolites such as antibiotics, lipopeptides, phenazines, and siderophores act as signal molecules to control microbial interactions, immune priming, and gene expression, which provides an antagonistic effect on microorganisms (Shao et al., 2024). These QS molecules, VOCs, and antimicrobial metabolites interact with each other to form an integrated chemical communication network, regulating plant immunity, microbial cooperation, and pathogen inhibition in rhizosphere triangular interactions (Jamil et al., 2022; Sibanyoni et al., 2026).

### 4.7 Medications such as lipopeptides and Signal Transduction Pathways

Lipopeptides are multifunctional microbial metabolite that directly inhibits microbial growth and act as an activator of plant defense signaling, hence, they are central regulators of plant-beneficial microbe-pathogen interactions (Ding et al., 2025). Surfactins, fengycins, iturins and viscosins are cyclic lipopeptides produced mainly by *Bacillus* and *Pseudomonas* species that disrupt the membrane of pathogens, inhibit spore germination, and suppress biofilm formation (Assena et al., 2024). In addition to these antimicrobial actions, they are microbe-associated molecular patterns (MAMPs) which trigger PTI (Ali J. et al 2024; Farace et al., 2015; Hudson et al., 2024).

Lipopeptides that are recognized by plasma membrane receptors trigger rapid influx of Ca^2+^, production of reactive oxygen species (ROS) and MAPK signaling, resulting in the activation of defense genes that are involved in cell wall reinforcement, synthesis of pathogenesis-related proteins and production of secondary metabolites (Escocard de Azevedo Manhães et al., 2021; Ma et al., 2024). Lipopeptide signaling also interacts with JA and ET signaling pathways to reinforce induced resistance (Jourdan et al., 2009). Furthermore, lipopeptides modulate quorum sensing, biofilms, and microbial competition, resulting in improved pathogen inhibition and rhizosphere stability (Zhao et al., 2026). As such, lipopeptides are antimicrobial molecules and immune system activators that connect microbial antagonism and plant immune activation and are important players in rhizosphere triangular warfare (Balleux et al., 2025). Additionally, recent research has demonstrated that lipopeptides produced from *Bacillus* can enhance systemic resistance against bacterial and fungal infections by activating JA/ET-associated defense signaling (Yang et al., 2023). All these metabolites create a chemically integrated communication network which regulates beneficial microbial recruitment and pathogen suppression in the rhizosphere (**Figure 4**).

## 5 Application of rhizosphere triangular warfare in plant protection

- Rhizosphere triangular warfare offers a mechanism to create sustainable plant protection strategies that combine plant immunity, beneficial microbes, and pathogen ecology (Chepsergon and Moleleki, 2023).
- Prescribed in this framework, however, is not only the use of chemical pesticides, but the use of naturally occurring biological interactions to increase disease suppression, crop robustness and rhizosphere stability.
- Beneficial microbes such as *Bacillus* spp., *Pseudomonas* spp., *Trichoderma* spp. and *Streptomyces* spp. suppress pathogens by different mechanisms, including antibiosis, lipopeptides, bacteriocins and volatile organic compounds (VOCs), iron chelating (siderophore), niche exclusion and enzymatic degradation of pathogen cell walls, which limit pathogen establishment and virulence (Zhou et al., 2021).
- These microorganisms trigger ISR, including JA- and ET-mediated pathways, to provide plants with more efficient and timely defense responses at little fitness cost (Yu et al., 2022).
- Plants also support these interactions by changing their exudation patterns, such as the secretion of sugars, organic acids, flavonoids, and coumarins, to specifically attract protective microbes and thus build a disease-suppressive rhizosphere microbiome (Du et al., 2025).
- Mechanistic understanding of these interactions has helped the development of microbial biocontrol agents, synthetic microbial consortia and microbiome-based soil management for increased functional stability and long-term disease suppression (Niu et al., 2020).
- The development of multi-omics, metabolomics and microbiome engineering has enabled the identification of keystone taxa, functional genes, and metabolic traits that dictate microbiome performance across a range of environmental conditions (Go et al., 2024).
- Real-world conditions expose plants and their associated microbiomes to a wide range of environmental elements, such as soil properties, climate, and seasonal variations, in contrast to laboratory-controlled trials (Singh et al., 2020).
- These factors affect the functionality and composition of microbial communities, making it more difficult to forecast and control plant-microbiome interactions consistently in various agricultural contexts (Berruto and Demirer, 2024; French et al., 2021).
- The discoveries open avenues for precision control of rhizosphere microbial communities, which can contribute to the development of environmentally friendly alternatives to chemical pesticides and promote sustainable and climate-resistant farming practices (Rezaee Danesh, 2025) (**Table 2**).
- Moving forward, future plant protection strategies are anticipated to combine the principles of microbiome engineering, synthetic ecology and precision agriculture to create rhizosphere communities that ensure the productivity of the crop in the face of the growing biotic and abiotic pressures.
- The rhizosphere triangular warfare concept is not only a conceptual approach to the interaction between plants, microorganisms and pathogens, but also a foundation for next-generation crop protection with the microbiome.

**Table 2:** Applications of rhizosphere triangular warfare (plant-beneficial microbe-pathogen interactions).

Application	Mechanistic basis	Role of beneficial microbes	References
Biological Disease Control in Crops	Antagonism, competition, and induced systemic resistance (ISR).	Beneficial microbes inhibit pathogens *via* antimicrobial compounds, niche competition, and induction of plant defenses.	Chhana, 2025; Singh et al., 2021
Induced Systemic Resistance (ISR)	Jasmonic acid (JA)/ethylene (ET) signaling, primed defense.	Plant growth-promoting rhizobacteria (PGPR)/plant growth-promoting fungi (PGPF) (e.g., *Pseudomonas*, *Trichoderma*) prime the plant immune system for broad-spectrum resistance without pathogen contact.	Pieterse et al., 2014
Microbiome Engineering for Crop Health	Root exudate recruitment, community assembly.	Manipulating rhizosphere communities to enrich protective microorganisms and suppress pathogens.	Li et al., 2021
Biofertilizer Development	Nutrient mobilization and growth-defense trade-off.	Beneficial microbes enhance nutrient uptake (Indole-3-acetic acid, 1-aminocyclopropane- carboxylate deaminase, siderophores) while aiding disease suppression.	Berg, 2009; Singh et al., 2022a, Singh et al., 2022b, Singh et al., 2022c
Integrated Pest Management (IPM)	Multiple suppression mechanisms.	Beneficial microbes incorporated into IPM reduce chemical pesticide dependency and enhance sustainable control.	Chhana, 2025
Stress Resilience Enhancement	Synergistic immune and physiological regulation.	Microorganisms help plants resist both biotic and abiotic stress by modulating immunity and stress responses.	Palkina et al., 2025
Post-Harvest Disease Mitigation	Surface colonization and induced defenses.	Microbial sprays reduce spoilage and pathogen growth on harvested produce.	Dehbi et al., 2023
Commercial Bioprotectants/Biopesticides	Microbial inoculants as products.	Beneficial strains developed into commercial products to protect crops sustainably.	Dehbi et al., 2023
Crop Quality and Yield Improvement	Growth promotion and disease suppression combined.	Improved yield and quality through PGPR/PGPF modulation of growth and immunity.	Berg, 2009

## 6 Future prospects

Mechanistic understanding and future advancements in rhizosphere triangular warfare will depend on the integration of emerging technologies with mechanistic understanding for the precision manipulation of plant-beneficial microbe-pathogen interaction (Trivedi et al., 2020). The combination of these three factors-artificial intelligence (AI), machine learning (ML), and multi-omics data-promises to enhance the prediction of microbial changes and disease outbreaks, as well as the positive functions of beneficial microbes, thereby enabling more effective data-driven microbiome engineering and precision crop management (Pace et al., 2025). Similarly, there are new opportunities to develop rhizosphere designer microbial consortia through genome engineering, the use of synthetic biology, nanotechnology-enabled delivery systems, and real-time rhizosphere biosensors that can manipulate rhizosphere processes to optimize root exudation and improve plant immunity (An et al., 2022; Ragland et al., 2024; Rodríguez-Leal et al., 2017; Webster et al., 2024).

Meanwhile, the recent development of spatial transcriptomics, spatial metabolomics, and single-cell RNA sequencing will allow us to dissect the spatiotemporal organization of microbial communities and to uncover cell-specific signaling networks controlling rhizosphere interactions in a changing environment (Li et al., 2025). These practices, in conjunction with the development of beneficial microbes that are resistant to climatic conditions, will enhance the crop’s ability to resist several biotic and abiotic stresses (Gahlot, 2026).

Although considerable progress has been made in understanding the rhizosphere, the transfer of laboratory findings to the field is plagued by several problems, as rhizosphere interactions are highly dependent on plant genotype, soil heterogeneity, environmental variations, and the complexity of the microbial community. Predictive modelling, long-term field validation, and incorporating the multi-omics data with ecological and agronomic data are therefore recommended for future studies to further increase the stability and reproducibility of microbiome-based interventions. All these advances together will speed up precision microbiome engineering and will lay the groundwork for sustainable crop protection and climate-smart agriculture (Rezaee Danesh et al., 2025; Trivedi et al., 2020).

## 7 Conclusion

The rhizosphere is a highly dynamic biological interface that is characterized by molecular and ecological interactions between plants, beneficial microbes and pathogens, which constantly influence the health of plants, plant disease resistance and ecosystem functioning. This review shows that rhizosphere triangular warfare is not driven by independent plant or microbial processes but by four elements of coordinated immune signaling, recruitment of the plant-associated microbiome by rhizosphere exudates, microbial competition, and pathogen counterstrategies. Virulence effectors and immune-suppressive mechanisms of pathogens cause changes to rhizosphere communities and overcome host defense, while beneficial microbes ISR, produce antimicrobial metabolites and mobilize nutrients for plant fitness. This results in the balance of these antagonistic forces dictating microbiome assembly, disease suppression and crop resilience.

The new opportunities unlocked by multi-omics, spatial biology and microbiome engineering are revolutionizing the way these interactions are understood, and new microbial consortia are designed which are functionally stable and contain keystone microorganisms. A combination of these mechanistic insights with predictive modelling, synthetic biology, and precision will help to pave the way for next-generation microbiome-assisted crop protection strategies. The idea of rhizosphere triangular warfare offers a single framework for the interactions between plants, microorganisms and pathogens, and lays the groundwork for sustainable and climate-smart farming through precision rhizosphere engineering.
